# Effects of dietary guanidinoacetic acid on growth performance, guanidinoacetic acid absorption and creatine metabolism of lambs

**DOI:** 10.1371/journal.pone.0264864

**Published:** 2022-03-11

**Authors:** Shiqi Zhang, Changjiang Zang, Jun Pan, Chen Ma, Caidie Wang, Xiaobin Li, Wenjie Cai, Kailun Yang

**Affiliations:** College of Animal Science, Xinjiang Agricultural University, Urumqi, China; University of Illinois, UNITED STATES

## Abstract

Guanidinoacetic acid (GAA) is the only precursor for the creatine synthesis of vertebrates. Creatine (Cr) and phosphocreatine (PCr) are able to provide energy for the rapid growth and development of the muscle tissue. This study evaluated the effects of dietary different levels GAA on growth performance, GAA absorption and creatine metabolism of lambs. Twenty-four 3-month-old healthy Kazakh male lambs (body weight = 27.35± 0.58 kg) were randomly divided into four groups with 6 lambs in each group. The lambs were fed with the basal diets supplemented with 0 (0 mg/kg group), 500 (500 mg/kg group), 1000 (1000 mg/kg group) and 1500 mg (1500 mg/kg group) GAA per kg diet (DM basis), respectively. The results showed that, as the GAA content of the diet increased, there was a quadratic change in DMI, with the lowest in the 500 mg/kg group and the highest in the 0 mg/kg group. The CK enzyme activity and ATP content in quadriceps muscle increased linearly with increasing levels of diary GAA in the diet. PCr levels and ADP levels in the longest dorsal muscle increased linearly with increasing levels of GAA in the diet. The relative expression of *SLC6A6* and *SLC6A8* mRNA in the jejunum and ileum mucosa showed a quadratic change as the dietary GAA level increased, with the lowest relative expression in both the 1500 mg/kg group. With the increase of dietary GAA level, both Cr concentration in hepatic vein plasma and the portal plasma GAA concentration shows a quadratic change, with the highest concentration in the 500 mg/kg group and the lowest concentration in the 0 mg/kg group. Therefore, dietary supplementation with 500~1000 mg/kg DM GAA is recommended for lambs.

## 1. Introduction

Guanidinoacetic acid (GAA) is the only precursor for the creatine synthesis of vertebrates. The exogenous addition of GAA in the diet can stimulate creatine biosynthesis in animal [1]. Creatine (Cr) and phosphocreatine (PCr) are key substances for energy transfer in vertebrate cells and form the hosphagen system. The phosphagen system increases the content of PCr and glycogen in muscles [2], providing energy for the rapid growth and development of muscle tissues [3, 4]. GAA has been used as performance-enhancing agent for monogastric animals. Dietary supplementation of 1.0 g/kg GAA can improve the carcass quality and energy metabolism of fattening pigs [5, 6]. Dietary supplementation of 0.6~1.2 g/kg GAA can promote growth performance of broiler [7, 8], and dietary supplementation of 1.2 g GAA can reduced the average daily intake [9]. Recently, the effect of GAA in ruminants has been gradually studied. Dietary addition of 0.6 or 0.9 g/kg DM GAA improved growth performance, nutrient digestion and ruminal fermentation in bulls [10]. However, the information concerning the dietary GAA supplementation on creatine metabolism and growth performance of lambs is limited.

During creatine biosynthesis, arginine: glycine amidotransferase (AGAT) catalyzes the reaction between arginine and glycine in kidneys to form GAA and ornithine. Then GAA enters the liver via blood transport and is methylated by S-adenosyl-L-methionine: N-guanidinoacetate methyltransferase (GAMT), with S-adenosylmethionine being the methyl donor to form Cr [11, 12]. The Cr is transported by the blood cotransported into cells via the Na+/Cl—dependent creatine transporter (solute carrier family 6 member 8, SLC6A8). GAA transport is also mediated by SLC6A8. Additionally, the SLC6A6 (solute carrier family 6 member 6, taurine transporter) and SLC6A13 (solute carrier family 6 member 13, γ-aminobutyric acid transporter 2) were also reported to be capable of transporting GAA [13, 14]. The SLC6A8, the expression in the small intestine is much higher than in large intestine, and over high substrate concentration inhibited its active expression [15–17]. Further investigation is needed for the effect of the activity of GAA-associated transporter on GAA absorption in the small intestine.

The GAA and Cr are amino acid derivatives of animal origin. Plant protein sources lack GAA and creatine [18]. GAA has been rarely been studied in sheep with a whole-plant protein diet. The effect of supplementation with GAA on performance, creatine metabolism and Changes in small intestine GAA-associated transporter activity in sheep needs to be confirmed. This study was conducted to investigate the effects of GAA on growth performance, GAA absorption and creatine metabolism of lambs.

## 2. Materials and methods

All experimental procedures involving animals were approved (animal protocol number: 2020024) by the Animal Welfare and Ethics Committee of Xinjiang Agricultural University, Urumqi, Xinjiang, China.

### 2.1 Experimental materials

Guanidinoacetic acid, without rumen protected treatment, was purchased from Gentech Biotechnology Co., Ltd, (Beijing, China), with a purity of over 98%.

### 2.2 Experimental design

Twenty-four 3-month-old healthy local Kazakh male lambs (*Ovis aries*, body weight = 27.35± 0.58 kg) from Xinjiang, China, were randomly divided into four groups with 6 lambs in each group. The lambs were fed with the basal diets supplemented with 0 (0 mg/kg group), 500 (500 mg/kg group), 1000 (1000 mg/kg group) and 1500 mg (1500 mg/kg group) GAA per kg diet (DM basis), respectively. A 7-d adaption period was followed by 100 d of experimental period. The basal diet was formulated according to the nutritional requirements of National Research Council version 2007 (NRC07) [19]. The feed compositions and nutrient levels of experimental ration are shown in Table 1.

**Table 1:** Feed ingredients and nutrient levels of experimental diets (%).

**Ingredient**	**Ratio**
Maize	30.00
Wheat bran	7.2
Soybean meal	12.00
Cottonseed meal	7.8
Alfalfa hay	20.00
Wheat straw	20.00
Premix^1^	3.00
Total	100
**Items**	**Nutrient levels (DM basis).**
Organism matter OM	89.87
Crude protein CP	16.58
Ether extract EE	1.53
Neutral detergent fiber NDF	46.16
Acid detergent fiber ADF	32.62
Calcium Ca	0.58
Phosphorus P	0.33
Lysine	0.81
Methionine+Cysteine	0.58

^1^ The premix provided the following per kg of the concentrate supplement: VA 10000.00 IU, VD3 2550.00 IU, VE 20.00 IU, niacin 20.00 mg, biotin 0.06 mg, Cu (as copper sulfate) 22.00 mg, Fe (as ferrous sulfate) 94 mg, Mn (as manganese sulfate) 80 mg, Zn (as zinc sulfate) 88 mg, I (as potassium iodide) 0.75 mg, Se (as sodium selenite) 0.5 mg, Co (as cobalt chloride) 0.33 mg, Ca (as calcium carbonate and calcium hydrogen phosphate) 0.35%, P (as calcium carbonate and calcium hydrogen phosphate) 0.125%, Nacl 0.8%.

### 2.3 Feeding management

The test lambs were numbered and housed in semi-open sheds with good ventilation (1.2 m × 1.5 m). The lambs were fed at 08:00 and 20:00 daily in 2 separate feedings. GAA was mixed into the concentrate for feeding. Lambs were fed and watered ad libitum. Feed intake was recorded daily and lamb weights were weighed every 15 days for each lamb. Dry matter intake (DMI), daily gain (ADG) and feed weight ratio (F:G) were calculated for each lamb. Disinfection, disinsection and immunization were conducted according to routine procedures of the farm.

### 2.4 Sample collection and processing

On 100^th^ day of the experiment, two and a half hours after the morning feeding, Lumianning anesthetic (the main component: xylazine hydrochloride, 0.1 g/sheep, Huamu Animal Health Care Products Inc. Jilin. China) was injected into the neck muscle of the lambs. After successful anesthesia, according to Pan et al. [20], jugular vein, hepatic vein and portal vein blood were taken at 4 h after the morning feeding. The collected blood samples were centrifuged at 1500 g and 4°C for 10 min to obtaine plasma. Plasma samples were collected in 1.5 mL microtubes and stored at -20°C.

After blood collection in portal vein and hepatic vein, the lambs were euthanized by exsanguination under anesthesia. The quadriceps and longissimus dorsi as well as hepatic tissue were isolated and stored in a sterile RNA-degrading enzyme-free cryopreservation tube and cryopreserved with liquid nitrogen immediately. After separating the small intestine (duodenum, jejunum and ileum) and removing the chyme, the small intestine was cleaned with physiological saline and dried with filter paper. The surface mucosa of each intestinal section was scraped, then put in sterile RNA-degrading enzyme-free cryopreservation tubes, respectively and cryopreserved with liquid nitrogen.

### 2.5 Determination of indicators

The diet samples were finely ground to pass through a 1-mm screen (Thomas-Wiley Laboratory Mill Model 4, Thomas Scientific USA, Swedesboro, NJ), and analyzed for dry matter (DM), organic matter (OM), crude protein (CP), fat (EE), acid detergent fibers (ADF), neutral detergent fibers (NDF), and amino acids (AAs). The 5.0 g samples were dried at 105°C overnight according to the AOAC(1990) method (925.09) [21]. Using a nitrogen analyzer combustion method (990.03; AOAC) (Model CNS-2000;LECO Company, St.1990), nitrogen content was determined, and CP was calculated as N× 6.25. Ether-like hexane extracts from the samples were determined in the Ankom Extraction System (Macedonia, New York). The contents of NDF and ADF were determined as described by Van Soest et al. [22] and Goering et al. [23]. The diet sample was analyzed after anaerobic hydrolysis by amino acid using a Sykam S433D amino acid Analyzer (Sykam, Germany).

The Cr and GAA in plasma were determined reference to the study by Wada et al. [24]. Firstly, 200 *μ*L of plasma was taken into another centrifuge tube, and 400 *μ*L of acetonitrile was added and mixed well to precipitate the protein. The mixture was put aside for 10 min and then centrifuged at 12000 *g* for 10 min. Subsequently, 100 *μ*L of clear supernatant was taken and mixed well with 200 *μ*L of phosphoric acid (2 mM). Then 20 *μ*L of the mixture was taken for the determination. Determination conditions: IC YS-50 weak acid cation exchange column (4.6 mm×125 mm) was used; the flow rate was 1.0 mL/min; the column temperature was 30°C; the detection wavelength was 210 nm; the elution mode was one-time linear elution; the sample size was 20 *μ*L.

The measurement of ATP and ADP in muscles: 300 mg of muscle tissue was taken into a 10-mL centrifuge tube, and 1.5 mL precooled 7% HClO_4_ solution was added. After 15-min soaking, the mixture was homogenized and then centrifuged at the rate of 12000 *g* at a low temperature for 10 min. Then 850 *μ*L of clear supernatant was taken, and its pH was regulated to 6.5 with 1 mol/L KOH solution. After 10-min soaking, the mixture was centrifuged at the rate of 12000 *g* at 4°C for 10 min. The clear supernatant was taken and filtered with a 45 *μ*m membrane for the determination. Chromatographic condition: The Waters SunFire C18 chromatography column (5 *μ*m, 250 mm×4.6 mm) was used; the mobile phase was the mixture of CH_3_OH and 50 mmol/L H_3_PO_4_ buffer solution with the volume ratio of 13.5: 86.5 (containing 2.5 mmol/L TBAHS; the pH being 7.00); the flow rate was 1.0 mL/min; the column temperature was 25°C; the sample size was 10 *μ*L; the UV detection wavelength was 254 nm [25].

The measurement of Cr and CrP in muscles: 900 *μ*L of the supernatant of the above tissue homogenate was taken, and its pH was adjusted to 6.5 by adding saturated K_2_CO_3_ solution. After 10-min soaking, the mixture was centrifuged at 12000 *g* at 4°C for 10 min. Then the supernatant of the mixture was filtered through a 45 *μ*m membrane for the determination. Chromatographic condition: The Agilent Zorbax ODS-C18 chromatography column (5 *μ*m, 250 mm×4.6 mm) was used; the mobile phase was the mixture of C_2_H_3_N and 29.4 mmol/L KH_2_PO_4_ buffer solution with the volume ratio of 2: 98 (containing 2.30 mmol/L TBAHS; the pH being 5.30); the flow rate was 1.0 mL/min; the column temperature was 30°C; the sample size was 20 *μ*L; the UV detection wavelength was 215 nm [9].

RNA extraction and Real-time PCR Total RNA was extracted using Trizol (Tiangen, Beijing, China). RNA concentration was measured with the NanoVue Plus spectrometer (Piscataway, New Jersey, USA). After the treatment with DNase I (Thermos Fisher Scientific), the first strand cDNA synthesis kit (RNA purified by Aidlab Biotechnologies Co., Ltd, Beijing, China) was used for reverse transcription. Primers for *SCL6A6*, *SCL6A8* and guanidinoacetate N-methyltransferase (*GAMT*) are shown in Table 2. The ABI 7300 Real-time PCR system (Stepone plus, ABI) was used. Reaction conditions: forty loops with each loop involving four processes, namely reaction at 95°C for 5 min, 30 s and 10 s and reaction at 60°C for 30 s. The RNA expression was calculated with the 2^-ΔΔCT^ method [26]. Selected samples in each process were analyzed in triplicate with GAPDH as the housekeeping gene.

**Table 2:** Sequences and parameters of primers for the real-time fluorescence quantitative PCR.

Gene name	Sequences of primers (5’-3’)	GenBank ID	Pruduct size/bp
*SLC6A8*	RP: TCCCCTACCTGTGCTACAAGA	101122930	78
	FP: GGATTCCTCCAATCAGGGCAA		
*SLC6A6*	RP: CATCGTGATCGTGTCCCTCC	101105033	147
	FP: AAGGTGTCCTCCAGGCAATG		
*GAMT*	RP: CAGGGGGTGTCCTCACCTA	101114955	78
	FP:TGATGACGAGCTTCCCGTTC		

GAMT = guanidinoacetate N-methyltransferase. SLC6A8 = solute carrier family 6 member 8, SLC6A6 = solute carrier family 6 member 6.

### 2.6 Data analysis

The data in this study were initially organized using Excel 2010 and then analyzed using GLM program of SAS software (Version 9.2; SAS Inst. Inc., Cary, NC, USA). The contrast coefficient of the orthogonal polynomial was used to determine the linear, quadratic and cubic effects of elevated GAA levels on different parameters. The results were presented as least squares means and standard error (SE), with *P*<0.05 indicating the effects are significant.

## 3. Results

### 3.1 Growth performance

As the GAA content of the diet increased, there was a quadratic change in DMI, with the lowest in the 500 mg/kg group and the highest in the 0 mg/kg group (Quadratic, *P*<0.01) (Table 3). However, the ADG and F:G were similar among four groups (*P*>0.05).

**Table 3:** Effects of guanidinoacetic acid supplementation on growth performance in lambs.

Item	GAA, mg per kg diet (DM)				SE	*P*-value		
	0	500	1000	1500		Linear	Quadratic	Cubic
Initial BW, kg	27.4	27.1	27.2	27.1	0.28	0.46	0.87	0.59
Day 100 BW, kg	42.8	42.4	43.3	43.1	0.42	0.49	0.83	0.19
DMI, g/day	1182	1146	1158	1165	4.94	0.08	<0.01	0.02
AG, kg	15.5	15.2	16.1	15.9	0.43	0.24	0.92	0.32
ADG, g	156.9	153.6	162.4	160.4	4.23	0.24	0.92	0.32
F:G	7.6	7.5	7.2	7.3	0.19	0.15	0.57	0.47

BW = body weight; DMI = dry matter intake; AG = average gain; ADG = average day gain; F:G = feed to gain ratio. The results were presented as least squares means and standard error (SE), with *P*<0.05 indicating the effects are significant. The least squares means for BW, DMI, ADG, F:G in the table are the results of an analysis of covariance with initial weight as the covariate. *n* = 6 lambs/group.

### 3.2 GAA and creatine concentration in jugular vein plasma

On 100 d of the experimental period, after 4 h of feeding, the GAA levels in the jugular vein plasma displayed quadratic changes with the increased GAA contents (Quadratic, *P*<0.05) (Table 4), with the highest in the 500 mg/kg group and the lowest in the 1500 mg/kg group. The jugular vein plasma creatine concentration was similar among four groups (*P*>0.05).

**Table 4:** GAA and creatine concentration in jugular vein plasma of lambs after feeding 4 h.

Item	GAA, mg per kg diet (DM)				SE	*P*-value		
	0	500	1000	1500		Linear	Quadratic	Cubic
GAA, *μ*mol/L	218.78	250.56	245.41	216.9	11.23	0.83	0.01	0.02
Creatine, *μ*mol/L	163.49	169.59	155.63	153.97	4.50	0.37	0.71	0.43

The results were presented as least squares means and standard error (SE), with *P*<0.05 indicating the effects are significant. *n* = 6 lambs/group.

### 3.3 The Cr-related metabolites in muscle tissues

The CK enzyme activity and ATP content in quadriceps muscle increased linearly with increasing levels of GAA in the die (Linear, *P*<0.05) (Table 5). PCr levels and ADP levels in the longest dorsal muscle increased linearly with increasing levels of GAA in the diet (Linear, *P*<0.05).

**Table 5:** Effects of supplementation with guanidinoacetic acid on creatine-related metabolism in lambs muscle.

Item	GAA, mg per kg diet (DM)				SE	*P*-value		
	0	500	1000	1500		Linear	Quadratic	Cubic
**Quadriceps femoris**								
Creatine kinase, U/g	3644.12	3639.79	3671.00	3676.43	7.97	<0.01	0.55	0.10
Cr, mmol/kg	27.99	21.41	24.83	29.28	2.95	0.57	0.08	0.51
PCr, mmol/kg	0.87	0.86	0.89	0.95	0.14	0.19	0.32	0.94
ATP, mmol/kg	3.29	3.01	3.61	3.67	0.14	0.01	0.26	0.04
ADP, mmol/kg	2.80	2.64	2.84	3.14	0.06	0.07	0.12	0.69
PCr:Cr, %	3.56	4.19	3.87	3.38	0.50	0.69	0.56	0.29
ADP: ATP ratio	0.85	0.88	0.81	0.85	0.06	0.83	0.91	0.43
**Longissimus dorsi**								
Creatine kinase, U/g	3660.72	3641.26	3630.23	3648.65	11.83	0.38	0.13	0.70
Cr, mmol/kg	33.54	34.72	31.93	34.98	1.99	0.87	0.65	0.28
PCr, mmol/kg	1.05	1.01	1.13	1.12	0.03	0.01	0.62	0.20
ATP, mmol/kg	4.65	4.91	5.13	5.32	0.21	0.03	0.85	0.99
ADP, mmol/kg	3.34	3.19	3.91	3.83	0.06	<0.01	0.80	0.02
PCr:Cr, %	3.23	2.94	3.61	3.20	0.20	0.84	0.67	0.02
ADP: ATP ratio	0.73	0.65	0.76	0.73	0.04	0.64	0.60	0.08

U is the symbol for one enzyme unit, one U is defined as the amount of the enzyme that produces a certain amount of enzymatic activity. mol/kg or U/g means the content in muscle protein. PCr = Creatine phosphate; Cr = creatine; The results were presented as least squares means and standard error (SE), with *P*<0.05 indicating the effects are significant. *n* = 6 lambs/group.

### 3.4 Contents of Cr in hepatic vein, and mRNA expression of GAMT in liver

With the increase of dietary GAA level, Cr concentration in hepatic vein plasma shows a quadratic change (Quadratic, *P*<0.05) (Table 6). with the highest Cr concentration in the 500 mg/kg group and the lowest Cr concentration in the 0 mg/kg group (*P*<0.05). However, the Cr concentration in the liver tissue did not show significant difference (*P*>0.05).

**Table 6:** Effects of supplementation with guanidinoacetic acid on creatine concentration in hepatic vein plasma and hepatic tissue of lambs.

Item	GAA, mg per kg diet (DM)				SE	*P*-value		
	0	500	1000	1500		Linear	Quadratic	Cubic
Hepatic tissue, *μ*g/g	103.90	117.41	108.92	105.83	7.54	0.94	0.28	0.43
Hepatic vein, *μ*mol/L	248.76	320.89	278.79	250.88	10.95	0.47	<0.01	0.02

The results were presented as least squares means and standard error (SE), with *P*<0.05 indicating the effects are significant. *n* = 6 lambs/group.

**Fig 1:**
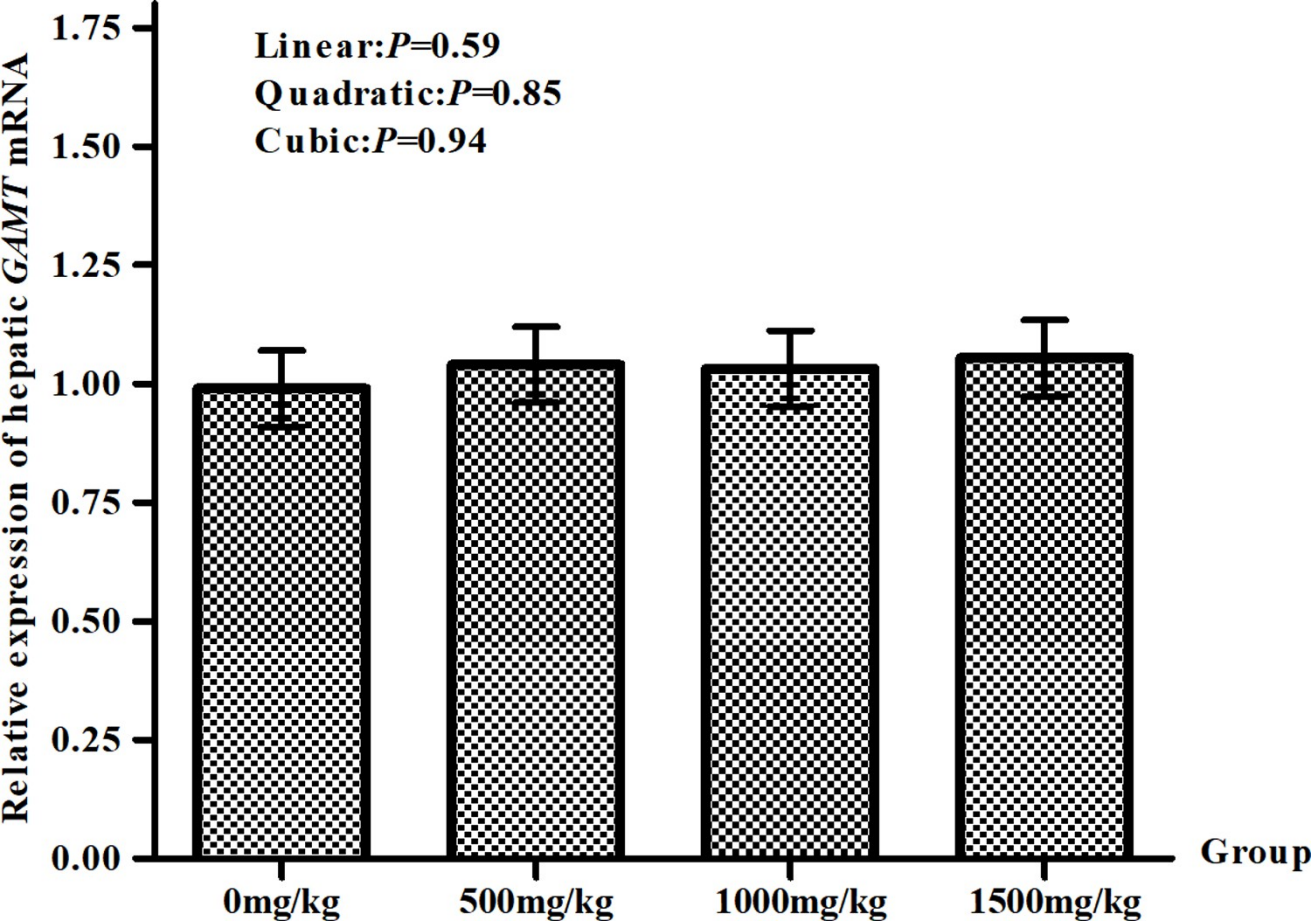
The mRNA expression of *GAMT* in lambs liver. The mRNA expression of GAMT in lambs live following a diet supplemented with 500, 1000, 1500 mg/kg DM GAA or no supplementation at 4 h after feeding. Values are least squares means ± SE (*n* = 5 lambs/group). The results were presented as least squares means and standard error (SE), with *P*<0.05 indicating the effects are significant.

After 4 h of the feeding, the mRNA expression levels of GAMT in GAA supplementation groups was increased compared with that in the 0 mg/kg group, but the values were similar (*P*>0.05) (Fig 1).

### 3.5 Portal plasma GAA concentration, contents of GAA and mRNA expression levels of SLC6A6 & SLC6A8 in small intestinal mucosa

After 4 h of feeding, GAA supplementation linearly increased the GAA concentration in duodenum mucosa (Linear, *P*<0.05) (Table 7). The GAA levels in the jejunum mucosa and ileum mucosa displayed quadratic changes with the increased GAA contents (Quadratic, *P*<0.05), and the GAA content was highest in the 1000 mg/kg group and the lowest GAA content in the 0 mg/kg group. The portal plasma GAA levels show quadratic changes with the increase of dietary GAA level (Quadratic, *P*<0.05). The GAA content was highest in the 500 mg/kg group and the lowest GAA content in the 0 mg/kg group.

**Table 7:** GAA concentration in portal plasma and in small intestinal segment mucosa of lambs after feeding 4 h.

Item	GAA, mg per kg diet (DM)				SE	*P*-value		
	0	500	1000	1500		Linear	Quadratic	Cubic
Portal vein, *μ*mol/L	236.12	273.98	269.22	245.83	10.46	0.61	<0.01	0.62
Duodnum, *μ*g/g	100.61	128.03	136.48	147.37	13.60	0.02	0.56	0.72
Jejunum, *μ*g/g	79.27	82.00	123.50	72.12	8.14	0.59	<0.01	<0.01
Ileum, *μ*g/g	85.82	103.65	122.15	98.48	7.38	0.10	0.01	0.21

The results were presented as least squares means and standard error (SE), with *P*<0.05 indicating the effects are significant. *n* = 6 lambs/group.

**Fig 2:**
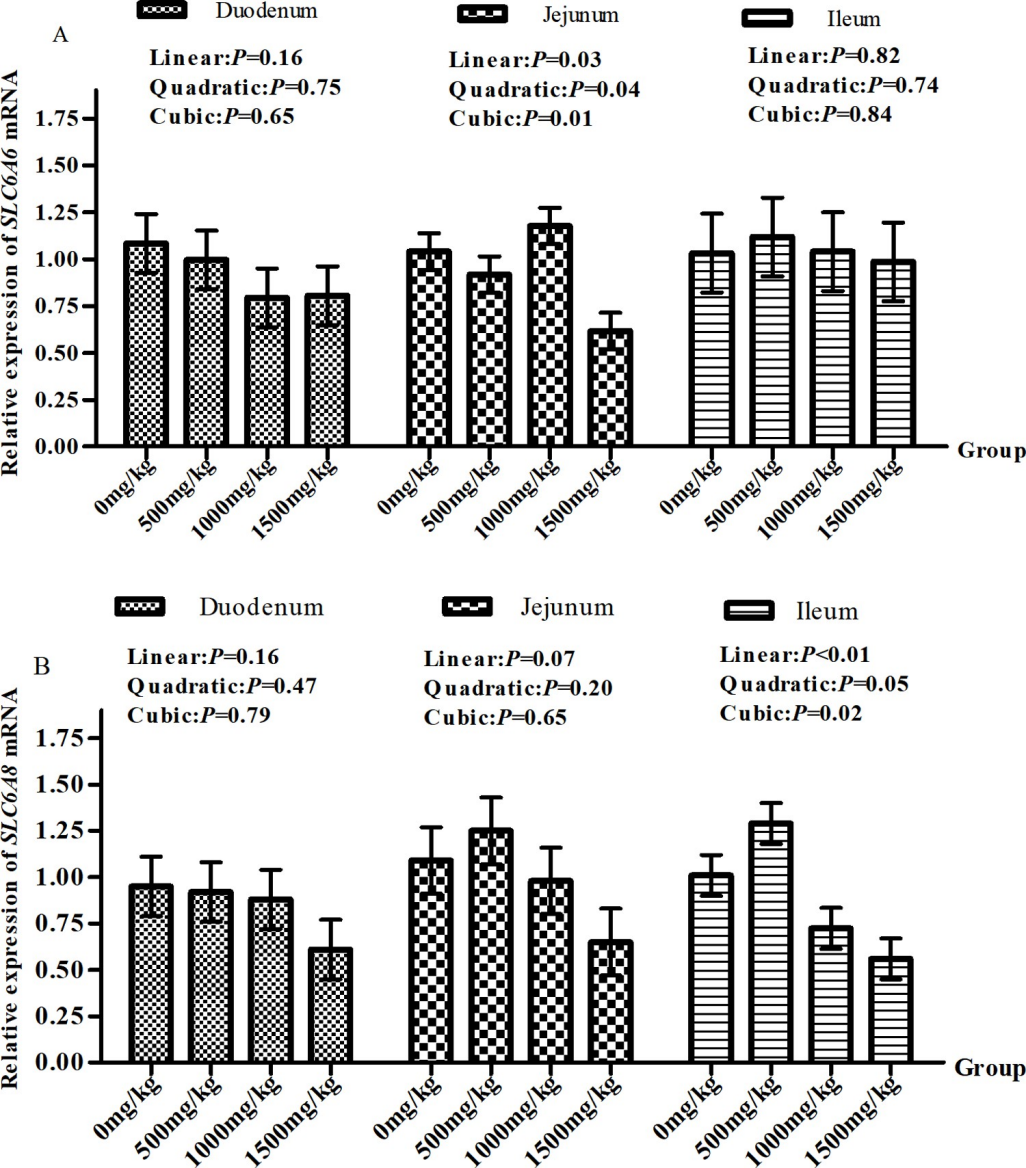
The mRNA expression levels of *SLC6A6*(A) & *SLC6A8*(B) in lambs small intestinal segment mucosa. The mRNA expression levels of *SLC6A6*(A) & *SLC6A8*(B) in lambs small intestinal segment mucosa following a diet supplemented with 500, 1000, 1500 mg/kg DM GAA or no supplementation at 4 h after feeding. Values are least squares means ± SE (*n* = 5 lambs/group).

The relative expression of *SLC6A6* and *SLC6A8* mRNA did not differ significantly in the duodenal mucosa(*P*>0.05) (Fig 2A and 2B). The relative expression of *SLC6A6* mRNA in the jejunum mucosa showed a quadratic change as the dietary GAA level increased, with the highest relative expression in the 1000 mg/kg group and the lowest relative expression in the 1500 mg/kg group(Quadratic, *P*<0.05) (Fig 2A). The relative expression of *SLC6A8* mRNA in jejunum mucosa decreased linearly with the increase of dietary GAA level (Linear, *P*<0.05) (Fig 2B), while the relative expression of *SLC6A8* mRNA in ileum mucosa showed a quadratic change with the increase of dietary GAA level, with the highest relative expression in the 500 mg/kg group and the lowest relative expression in the 1500 mg/kg group (Quadratic, *P*<0.05) (Fig 2B).

## 4. Discussion

Dietary supplementation of 0, 500, 1000 and 1500 mg/kg DM GAA, there were quadratic changes in DMI with the 500 mg/kg group DMI was lowest. The GAA supplementation reduced the DMI of lambs. The reason for this effect may be that dietary GAA improves phosphorocreatine levels and increased ATP content in the muscle. Both creatine and phosphocreatine help replenish ATP from ADP via the CK reaction to maintain ATP at a constant level [27–29]. Dietary 1000 mg/kg GAA increased creatine kinase (CK) levels in quadriceps as well as adenosine diphosphate (ADP) and PCr in Longissimus dorsi, suggesting promoting the CK response in lambs muscle [30]. Increase of creatine phosphate levels saves raw materials and energy substances for protein synthesis, and energy supply beyond requirements so that it reduces food intake [31]. The promotion effect of creatine synthesis of GAA has also been found on broiler and bullfrog fed with plant protein basal diet [32–35]. Dietary supplementation of 1.2 g GAA can reduce the average daily intake [9]. In this study, the ADG of lambs was similar. He et al. [36] has found a similar phenomenon that dietary GAA can promote the growth performance of growing-finishing pigs in the early period and has no significant effect in the late stage. Besides, according to changes in the GAA level in portal vein blood the body weight growth may be associated with GAA absorption in lambs. Speer et al. [37] reported that the bioavailability of GAA in cattle was about 50%. Li et al. [10] reported that the supplementation with 0.6 g/kg DM GAA in bull diets increased the proportion of Vibrio fibrate, Vibrio tumefaciens and Bacillus rumenophilus, increased cellulase, pectinase and protease activities and increased total volatile fatty acid concentrations in bulls. Therefore, the dietary supplementation with GAA may also affect growth by influencing rumen microbial metabolism, which is lacking in this study and needs to be further investigated.

The Cr enters blood circulation from the hepatic vein and then enters cells via transporters for phosphorylation metabolism. In this study, the Cr content in the hepatic vein of the 500 mg/kg group is highest, indicating that the dietary GAA promotes creatine synthesis. The expression of *GAMT* mRNA was similar in liver and it is possible that the portal plasma GAA concentrations in all groups exceeded the *Km* (98 *μ*mol/L) of GAMT [38]. Furthermore, a quadratic effect was observed with highest concentration observed with 500 mg/kg of GAA in hepatic vein plasma Cr concentration, which is supported by He et al. [36], where dietary 300 mg/kg GAA tail vein serum GAA being the highest level not 600 mg/kg and 900 mg/kg. The reason they have proposed may be that the expression of *AGAT* was reduced because of the feedback mechanism of GAA synthesis. *AGAT* is weakly expressed and does not play a significant role in GAA synthesis in the liver [39]. Based on the portal vein GAA changes and hepatic vein creatine changes, we speculate that 1500 mg/kg dietary GAA (2.4 g/day, approximately 60 mg/kg BW) can inhibit the absorption and transport of GAA in the gastrointestinal tract and the amount of GAA transported from the portal vein to the liver decreases of lambs at the late stage of the experiment.

GAA in the diet is absorbed by the gut through the portal blood into the liver for creatine synthesis. The GAA transporter *in vivo* including SLC6A8, SLC6A6 and SLC6A13 have been reported [13, 14]. The SLC6A8, the expression in the small intestine is much higher than in large intestine, and has an inhibiting effect at high substrate concentrations [15–17]. In this study, supplemental GAA, especially 500 mg/kg GAA, tended to increase plasma GAA in portal vein and, but 1500 mg/kg and 1000 mg/kg group not done yet. It is possible that gastrointestinal contents containing higher concentrations of GAA may inhibit the functional expression of relevant transporters in small intestine epithelial cells. This speculation was also confirmed by the varying degrees of reduced expression levels of *SLC6A8* and *SLC6A6* mRNA in the jejunum mucosa in the 1500mg/kg group. Therefore, GAA entering the liver is not linearly increased with the increase of the dietary GAA in the late stage of the experiment. The appropriate amounts of dietary GAA at different growth phases of lambs need further investigation.

## 5. Conclusions

In summary, at dietary supplementation of 0, 500, 1000 and 1500 mg/kg DM GAA, there were quadratic changes in DMI with the 500 mg/kg group DMI was lowest. And a linear increase in CK activity and ATP levels in the quadriceps and a linear increase in PCr levels in the longest dorsal muscle as the dietary GAA dose increased. However, a quadratic effect was observed with lowest relative expression of SLC6A8 mRNA observed with 1500 mg/kg of GAA. The highest portal plasma GAA concentration with a quadratic effect was observed with in 500 mg/kg DM GAA group. Therefore, dietary supplementation with 500~1000 mg/kg DM GAA is recommended for lambs.

## Supporting information

S1 Table Feed ingredients and nutrient levels of experimental diets (%). (XLS)

S2 Table Sequences and parameters of primers for the real-time fluorescence quantitative PCR. (XLSX)

S3 Table Effects of guanidinoacetic acid supplementation on growth performance in lambs. (XLSX)

S4 Table GAA and creatine concentration in jugular vein plasma of lambs after feeding 4 h. (XLSX)

S5 Table Effects of supplementation with guanidinoacetic acid on creatine-related metabolism in lambs muscle. (XLSX)

S6 Table Effects of supplementation with guanidinoacetic acid on creatine concentration in hepatic vein plasma and hepatic tissue of lambs. (XLSX)

S7 Table GAA concentration in portal plasma and in small intestinal segment mucosa of lambs after feeding 4 h. (XLSX)

S8 Table The mRNA expression of GAMT in lambs liver. (XLSX)

S9 Table The mRNA expression levels of SLC6A6(A) & SLC6A8(B) in lambs small intestinal segment mucosa.
(XLSX)
